# Magnetic Material Assessment of a Novel Ultra-High Step-Up Converter with Single Semiconductor Switch and Galvanic Isolation for Fuel-Cell Power System

**DOI:** 10.3390/ma10111311

**Published:** 2017-11-15

**Authors:** Chih-Lung Shen, Heng Liou

**Affiliations:** Department of Electronic Engineering, National Kaohsiung First University of Science and Technology, Kaohsiung 82445, Taiwan; u0452809@nkfust.edu.tw

**Keywords:** fuel cells, magnetic material, single semiconductor switch, ultra-high step-up converter

## Abstract

In this paper, a novel step-up converter is proposed, which has the particular features of single semiconductor switch, ultra-high conversion ratio, galvanic isolation, and easy control. Therefore, the proposed converter is suitable for the applications of fuel-cell power system. Coupled inductors and switched capacitors are incorporated in the converter to obtain an ultra-high voltage ratio that is much higher than that of a conventional high step-up converter. Even if the turns ratio of coupled inductor and duty ratio are only to be 1 and 0.5, respectively, the converter can readily achieve a voltage gain of up to 18. Owing to this outstanding performance, it can also be applied to any other low voltage source for voltage boosting. In the power stage, only one active switch is used to handle the converter operation. In addition, the leakage energy of the two couple inductors can be totally recycled without any snubber, which simplifies the control mechanism and improves the conversion efficiency. Magnetic material dominates the conversion performance of the converter. Different types of iron cores are discussed for the possibility to serve as a coupled inductor. A 200 W prototype with 400 V output voltage is built to validate the proposed converter. In measurement, it indicates that the highest efficiency can be up to 94%.

## 1. Introduction

The power generation coming from petro-chemical, coal, and liquefied natural gas (LNG) results in a series of damages like environmental destruction, climate change, and aerial contamination. Therefore, renewable resources have been growing rapidly in recent years to overcome the mentioned problems. Nowadays, green energy is adopted in many fields from household use to industry application. In some applications, such as electric vehicle (EV) and grid-tied renewable-energy system, a high DC voltage to be in the range of 380 to 420 V is required [1]. Accordingly, incorporating a high step-up DC/DC converter to boost a low-voltage distributed energy resource becomes essential in green energy system.

A high step-up converter developed for photovoltaic (PV) module, wind turbine, or fuel cells is able to boost up a low voltage to a much higher level [2,3,4,5,6,7,8,9,10], thus, which can accomplish power injecting into a DCbus [11,12]. Magnetic inductor is a main component that is much more important than any other devices in a high step-up converter. A theoretical model of magnetically coupling inductor is studied to analyze the saturation condition [2]. Utilizing parallel paths to share input current for higher power processing is proposed in [3,4]. An extra high gain can be achieved by means of voltage multipliers, which has been presented in [5]. Some researchers derive new topologies from boost-type configuration [6,7], while the literature [8] concentrates on interleaved operation and leakage energy recycling. In [9], an isolated step-up converter based on quasi Z-source is presented to lower the component count. Combination of boost configuration, coupled inductor and interleaving control is discussed in [10]. For the achievement of high voltage gain, the method that a high step-up converter incorporates coupled inductor [13] and/or switched capacitor [14] is a common approach. However, the shortcomings of galvanic isolation, more active switches, and without high enough voltage gain for direct grid-tied application still exist. For this kind of converter, magnetic device, that is, the coupled inductor, will dominate the power conversion performance of a high step-up converter. The characteristics of a magnetically coupling component should be discussed. Unfortunately, which type of iron core should be feasible and adopted is missed in most literature.

Magnetic core, which includes Ferrite, Molypermalloy Powder (MPP), High Flux, and Kool Mμ, are widely used in switching-mode power converters. The characteristics of these magnetic materials, such as flux density, biasing capability, permeability, and core loss, are different. The core loss is of vital importance in power converter design. The following literature discussed core loss from various aspects. A simplified model is proposed in [15] to estimate core loss, which considered the effects of both frequency and temperature. Another one by means of investigating the toroidal cores of Ferrite material explored core loss [16]. In [17], the authors introduced a solution to calculate the core loss of a transformer in switching the power supply with realistic measurements. As for non-sinusoidal input, the literature [18] presented the core loss estimation covering the influences of frequency and duty cycle.

**Figure 1:**
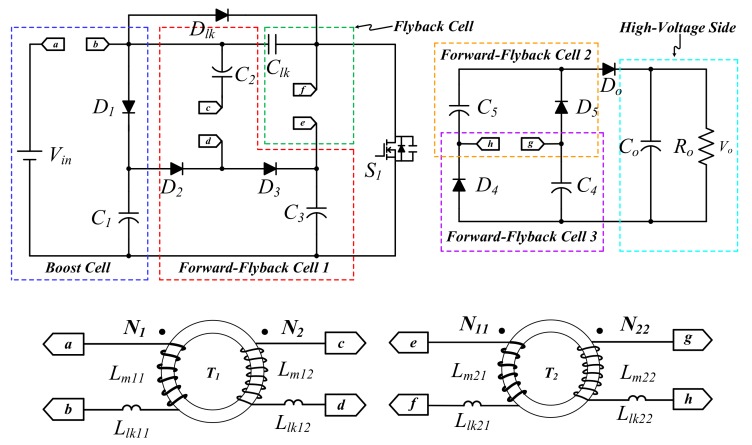
The main power stage of the proposed single-switch isolated ultra-high step-up converter (SIUSC).

This paper proposes a single-switch isolated ultra-high step-up converter (SIUSC), which can achieve an ultra-high voltage gain for low-voltage input. Its voltage conversion ratio is much higher than that of any other step-up converter. In addition, the SIUSC inherently has the particular features of only one semiconductor switch needed, galvanic isolation, being suitable for any low voltage source, and easy control. In order to characterize a better performance, different materials of magnetic cores are considered and then valued. The converter power stage is depicted in Figure 1. The SIUSC includes several parts: one boost cell, three forward-flyback cells, and one flyback cell, in which the coupled inductor *T*_2_ performs galvanic isolation. For a better understanding, the advantages of the proposed SIUSC are summarized as follows:

(1) Only one active switch is utilized.
(2) The energy stored in leakage inductance can be recycled.
(3) The voltage conversion ratio is high enough so that SIUSC is capable of dealing with low voltage input.
(4) The structure of the SIUSC has galvanic isolation.

This paper is organized as follows. After the introduction in Section 1, Section 2 details the steady-state analysis of the proposed converter followed by the voltage gain derivation is presented in Section 3. After the inductance design of the coupled inductor operated in continuous conduction mode (CCM) in Section 4, the evaluation of coupled-inductor loss for different magnetic materials is given in Section 5, providing an appropriate selection of magnetic core. Finally, the experimental results measured from a 200-W prototype are given in Section 6 to validate the proposed converter, while the conclusion is summarized in Section 7.

## 2. Operation Mode of the Proposed Converter

Figure 1 is the configuration of the converter, in which symbol definitions are summarized in the following. The *V_in_* and *V_o_* signify the terminal voltages at low- and high-voltage sides, respectively. *S*_1_ is the semiconductor switch, while *D*_1_–*D*_5_, *D_lk_*, and *D_o_* are power diodes. *C*_1_–*C*_5_, *C_lk_*, and *C_o_* are capacitors that are employed in the power stage. The magnetically-coupled device *T*_1_ has *N*_1_ turns at the primary, *N*_2_ turns at the secondary, magnetizing inductances *L_m_*_11_ and *L_m_*_12_, and leakage inductances *L_lk_*_11_ and *L_lk_*_12_. Similarly, *N*_11_ and *N*_22_ represent the primary and the secondary turns of *T*_2_, *L_m_*_21_ and *L_m_*_22_ denote the primary and secondary magnetizing inductances, respectively, and *L_lk_*_21_ and *L_lk_*_22_ express the leakage inductances. Before the description of converter operation, some assumptions are made as follows:

(1) In Figure 1, all of the coupled inductors are in CCM.
(2) Parasitic input capacitance of the main switch, *C_iss_*, is neglected, and all of the diodes are considered ideal.
(3) The capacitances of all capacitors are considered large enough to ignore the ripples across them. Thus, their voltage can be regarded as constant during one switching period.
(4) The turn ratios of *T*_1_ and *T*_2_ are both equal to *n*. That is, *n*_1_ = *n*_2_ = *N*_2_/*N*_1_ = *N*_22_/*N*_11_.

**Figure 2:**
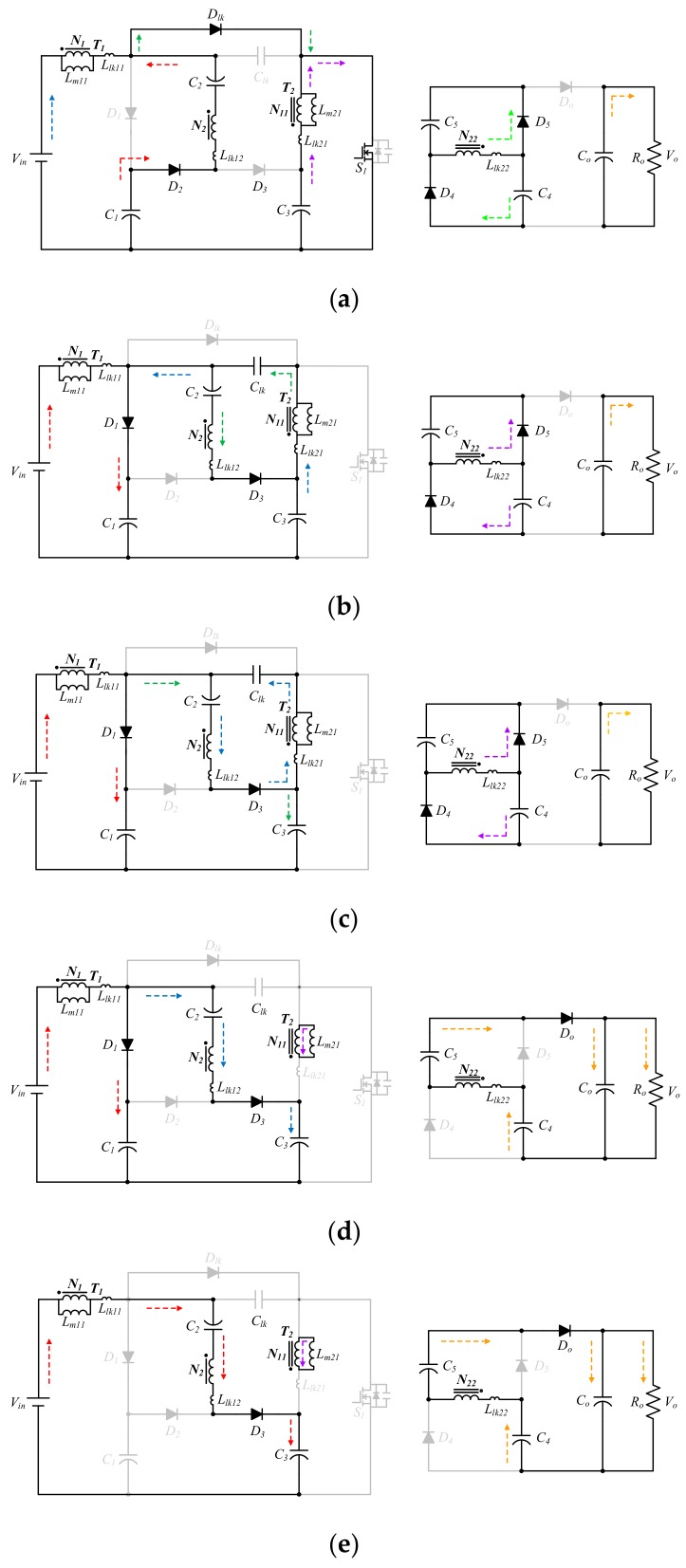

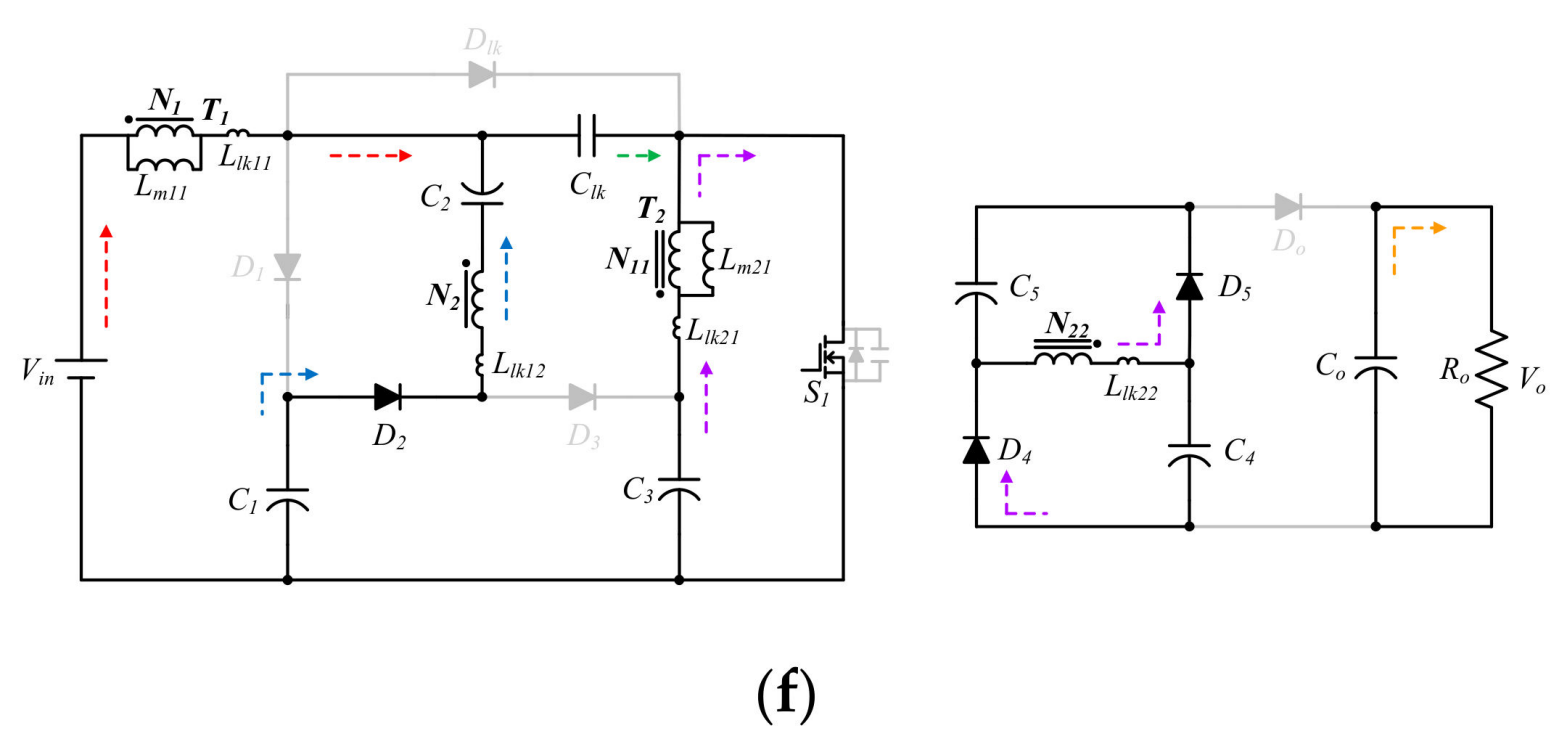
Mode equivalents of the proposed SIUSC over one switching cycle. (**a**) Mode 1 [*t*_0_, *t*_1_]; (**b**) Mode 2 [*t*_1_, *t*_2_]; (**c**) Mode 3 [*t*_2_, *t*_3_]; (**d**) Mode 4 [*t*_3_, *t*_4_]; (**e**) Mode 5 [*t*_4_, *t*_5_]; and (**f**) Mode 6 [*t*_5_, *t*_6_].

**Figure 3:**
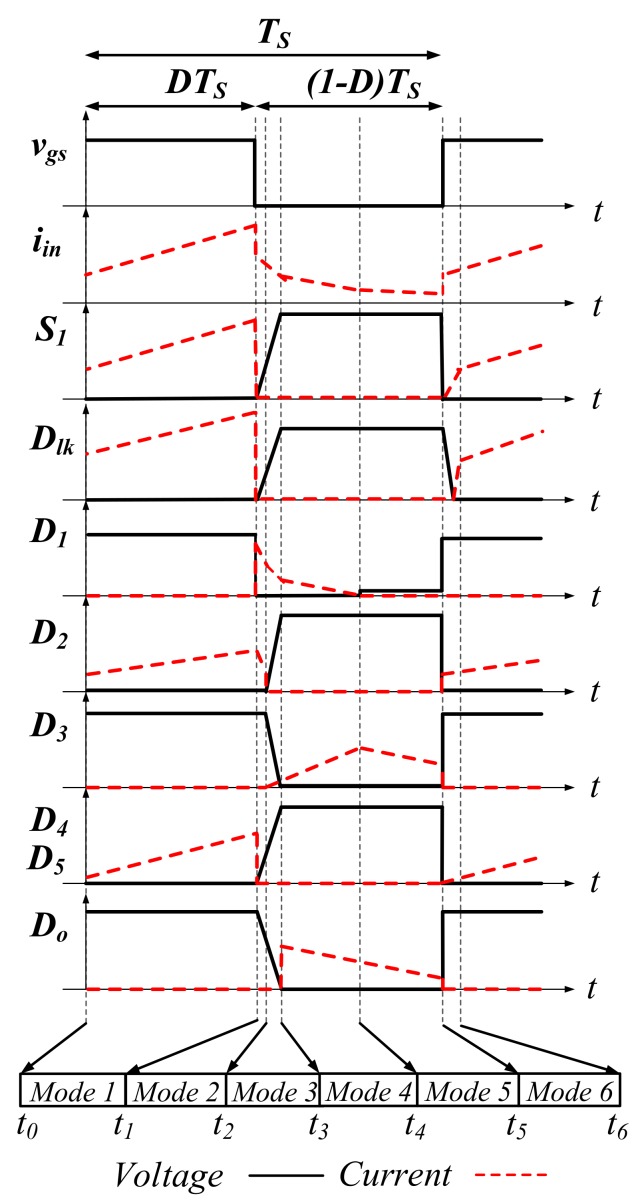
The key waveforms of the proposed SIUSC.

The operation of the proposed SIUSC can mainly be divided into six operation modes, whose corresponding equivalents are represented in Figure 2. Key waveforms of the SIUSC over one switching cycle are depicted in Figure 3. The operation of the converter is described mode by mode in the followings.

**Mode 1 [*t*_0_, *t*_1_] (see Figure 2a):** *The converter operation over one switch cycle starts at Mode 1, in which the switch S_1_ is turned ON at t = t_0_. The magnetizing inductors L_m11_ and L_m21_ absorb energy from V_in_ and C_4_, respectively. Meanwhile, capacitor C_1_ and the secondary of T_1_ release energy to capacitor C_2_ via D_2_ and S_1_. Once the switch S_1_ is turned OFF, operation of the converter enters into the next mode.*
**Mode 2 [*t*_1_, *t*_2_] (see Figure 2b):** *In Mode 2, switch S_1_ is in OFF state. During this time interval, the energy of L_lk11_ is forwarded to capacitor C_1_ via diode D_1_. Similarly, the energy of L_lk21_ is dumped to C_lk_ via D_3_. When i_D2_ drops to zero, this mode ends.*
**Mode 3 [*t*_2_, *t*_3_] (see Figure 2c):** *The S_1_ remains the same status as in Mode 2. The energy of L_m11_ is forwarded to capacitor C_1_. Meanwhile, the capacitor C_3_ is charged via the loop of V_in_-N_1_-C_2_-N_2_-D_3_. When the capacitor C_lk_ stops absorbing energy from L_lk21_, this mode ends.*
**Mode 4 [*t*_3_, *t*_4_] (see Figure 2d):** *During the interval of Mode 4, switch S_1_ is in OFF state. Input voltage V_in_ and magnetizing inductance L_m11_ proceed with energy releasing toward C_1_ and C_3_ via D_1_ and D_3_, respectively. In addition, the L_m21_ begins pumping its energy to output capacitor C_o_ via the loop of C_4_-N_22_-C_5_-D_o_-C_o_. Since C_4_ and C_5_ are in series at this energy-pumping loop, both capacitors also forward their energy to C_o_. The current following through D_1_, i_D1_, decreases. Mode 4 ends when i_D1_ is equal to zero.*
**Mode 5 [*t*_4_, *t*_5_] (see Figure 2e):** *The switch has the same status as in Mode 4. Since the capacitor C_1_ has been fully charged, the L_m__11_ and L_m__21_ pump energy to C_3_ and output, respectively. This mode will end as current i_Do_ falls to zero.*
**Mode 6 [*t*_5_, *t*_6_] (see Figure 2f):** *In this mode, switch S_1_ is in ON-state again. The energy stored in capacitor C_lk_ is drawn out via S_1_; that is, the leakage energy of L_lk__21_ is successfully recycled. This mode ends when i_Dlk_ starts to increase, and converter operation over one switching cycle is completed.*

## 3. Voltage Gain Derivation

In this section, the voltage conversion ratio of SIUSC will be derived. For high power applications, the SIUSC is designed to operate in CCM. Furthermore, the assumptions made in the previous section are also considered. In addition, the phenomenon that occurs during switching transient is ignored.

It can be found that the output voltage *V_o_* is equal to the sum of *V_C_*_4_, *n*_2_*V_Lm_*_21_, and *V_C_*_5_. Hence, in order to determine the voltage ratio of *V_o_* to *V_in_*, the voltages of *V_C_*_1_, *V_C_*_2_, *V_C_*_3_, *V_C_*_4_, *V_C_*_5_, *V_Clk_,* and *V_Co_* have to be obtained in advance. Firstly, the voltage across *C*_1_ can be determined by applying volt-second balance criterion (VSBC) to *L_m_*_11_, which can yield.

(1) $$V_{C1}\;=\;\frac{1}{1-D}V_{in}.$$

As for *V_C_*_2_, the voltage across *C*_2_ is the sum of *V_C_*_1_ and *n*_1_*V_Lm_*_11_ when *S*_1_ is in ON state. Thus, *V_C_*_2_ can be found by

(2) $$V_{C2}\;=\;\frac{1\;+\;n_{1}(1\;-\;D)}{1\;-\;D}\;V_{in}.$$

The *V_C_*_3_ is equal to the series voltage of *V_in_*, *V_Lm_*_11_, *n*_1_*V_Lm_*_11_, and *V_C_*_2_, accordingly, which can be

(3) $$V_{C3}\;=\;\frac{2\;+\;n_{1}}{1\;-\;D}\;V_{in}.$$

The capacitors *C*_4_ and *C*_5_ are charged simultaneously while *S*_1_ is closed. Thus, both voltages *V_C_*_4_ and *V_C_*_5_ are identical and equal to *n*_2_*V_Lm_*_21_, and then the following relationship holds:

(4) $$V_{C4}\;=\;V_{C5}\;=\;\frac{n_{2}(2\;+\;n_{1})}{1\;-\;D}\;V_{in}.$$

With respect to the voltage across capacitor *C_lk_*, the voltage *V_Clk_* can be determined from *V_Lm_*_12_, *V_C_*_2_ and *V_Lm_*_21_. It is given by

(5) $$V_{Clk}\;=\;\frac{1\;+\;n_{1}\;+\;D}{{\left(1\;-\;D\right)}^{2}}\;V_{in}.$$

Since the output voltage *V_o_* = *V_C_*_4_ + *V_C_*_5_ − *n*_2_*V_Lm_*_21_, the voltage *V_Co_* can be determined from

(6) $$V_{Co}\;=\;V_{o}\;=\;(\frac{2\;+\;n_{1}}{1\;-\;D}n_{2}\;+\;\frac{2\;+\;n_{1}}{1\;-\;D}n_{2}\;-\;\frac{D(2\;+\;n_{1})}{1\;-\;D}n_{2})\;V_{in}$$

Once the voltages *V_C_*_1_, *V_C_*_2_, *V_C_*_3_, *V_C_*_4_, *V_C_*_5_, *V_Clk_* and *V_Co_* are given in terms of *V_in_*, the conversion ratio of output to input voltages, *V_o_*/*V_in_*, can readily be found by

(7) $$\frac{V_{o}}{V_{in}}\;=\;\frac{n_{2}(2\;+\;n_{1})(2\;-\;D)}{{\left(1\;-\;D\right)}^{2}}$$

## 4. Inductance Design for CCM

The boundary condition of *i_Lm_*_1_ is designed at 20% of full load for overall efficiency consideration. That is, the output resistance is 4000 Ω at boundary. The maximum and minimum currents of *i_Lm_*_1_, denoted as *I_Lm_*_1*,max*_ and *I_Lm_*_1*,min*_, respectively, can be calculated by

(8) $$I_{Lm1,max}\;=\;I_{Lm1,avg}\;+\;\frac{\Delta i_{Lm1}}{2}$$

and

(9) $$I_{Lm1,min}\;=\;I_{Lm1,avg}\;-\;\frac{\Delta i_{Lm1}}{2},$$

where *I_Lm_*_1*,avg*_ is the average value of *i_Lm_*_1_ and ∆*i_Lm_*_1_ stands for the current change on the magnetizing inductance over switch ON or OFF interval.

To make sure the magnetizing inductance *L_m_*_1_ is in CCM, the current *I_Lm_*_1*,min*_ should be greater than zero. Additionally, the ∆*i_Lm_*_1_ and *I_Lm_*_1*,avg*_ can be determined as follows:

(10) $$\Delta i_{Lm1}\;=\;\frac{v_{Lm1}}{L_{m1}}DT_{s}\;=\;\frac{V_{o}{\left(1-D\right)}^{2}D}{f_{s}L_{m1}(2-D)(2+n_{1})n_{2}},$$

and

(11) $$I_{Lm1,avg}\;=\;\frac{(2-D)(2+n_{1})n_{2}}{{\left(1-D\right)}^{2}}\;I_{o},$$

where *f_s_* represents the switching frequency in hertz. Thus,

(12) $$I_{Lm1,min}\;=\;\frac{(2-D)(2+n_{1})n_{2}}{{\left(1-D\right)}^{2}}\;I_{o}\;-\;\frac{\Delta i_{Lm1}}{2}\;=\;0,$$

By rearranging the Equations (9)–(12), the minimum value that makes coupled inductor *T*_1_ operate in CCM is obtained as:

(13) $$L_{m1}\;=\;\frac{D{\left(1-D\right)}^{4}R_{o}}{2f_{s}n_{2}{}^{2}{\left(2+n_{1}\right)}^{2}{\left(2-D\right)}^{2}}$$

## 5. Magnetic Core Selection

There are three types of toroidal cores, MPP, High Flux, and Kool Mμ, which are widely adopted in switching power supply. They are also considered to serve as the magnetic core of the coupled inductors in SIUSC and are discussed in this section. Based on (13), if given that *D* = 0.47, *f_s_* = 50 kHz, *n*_1_ = *n*_2_ = 1, and *R_o_* = 4 kΩ, the inductance of *L_m_*_1_ will be 70.4 μH. In addition, in order to process a power of 200 W, the *I_Lm_*_1*,avg*_ should be 8.33 A. At this situation, current ripple Δ*i_Lm_*_1_ is limited within 2 A. As a result, the maximum current of *L_m_*_1_, *I_Lm_*_1*,max*_ is calculated as

(14) $$I_{Lm1,}{}_{\max}\;=\;I_{Lm1,avg}\;+\;\frac{\Delta i_{Lm1}}{2}\;=\;9.33\;A$$

The maximum increment on flux density of a specific magnetic material, Δ*B_max_*, has to be known to avoid a coupled inductor from going saturation. The Δ*B_max_* can be found from the manufacturer’s datasheet. With the values of maximum magnetizing current, maximum flux change, switching frequency, and volume of core, it can benefit a designer to assess the loss of a coupled inductor and then to choose an appropriate magnetic core [19].

### 5.1. MPP Core

**Figure 4:**
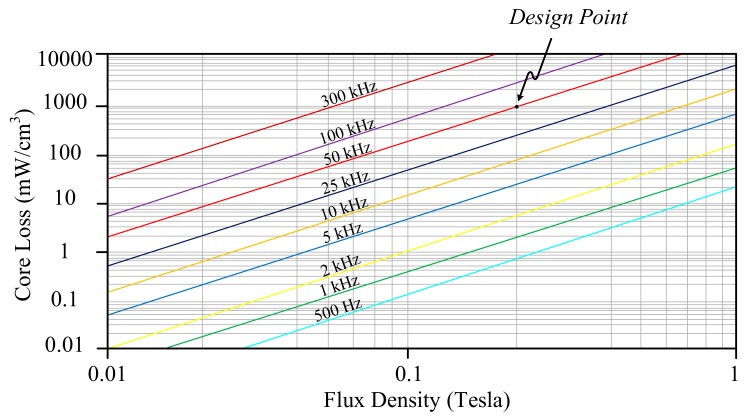
The relationship between core loss and flux density of Molypermalloy Powder (MPP) while permeability equals 125 μH/m.

The core loss of MPP can be calculated by

(15) $$P_{core\;loss-MPP}\;=\;53.05\;B^{2.06}f_{s}{}^{1.56},$$

in which the *B* is flux density and *f_s_* is operation frequency. A corresponding plot is shown in Figure 4, in which the permeability equals 125 μH/m. In general, the *B* will fall within the range from 0.2 to 0.25 Tesla. If an MPP core with part No. 55324-A2, whose volume is 6.088 cm^3^, is considered, the associated power loss density will be 861.39 mW/cm^3^ under the design conditions that *B* is given as 0.2 Tesla and *f_s_* = 50 kHz. As a result, the core loss is computed as 5.244 W.

Based on the given values of *L_m_*_1_, *I_Lm_*_1*,max*_, *B*, and the effective cross-section area of 0.678 cm^2^ for MPP 55324-A2, the turns of primary side can be determined as follows:

(16) $$N_{MPP}\;=\;\frac{L_{m1}\Delta I_{max}}{\Delta B_{max}A_{e}}\;\times \;10^{-2}\;=\;\frac{70.4\times 9.33}{0.375\times 0.678}\;\times \;10^{-2}\;=\;25.83$$

Since turns ratio should be an integer, *N_MPP_* is chosen as 26 turns. An average length of one turn is 3.44 cm, which yields the total length of the winding at the primary is

(17) $$l_{MPP}\;=\;26\;\times \;3.44\;=\;894.4\;\mathrm{mm}$$

A copper wire with cross-section area of 0.518 mm^2^ is adopted for a maximum carried current of 11 A. The resistivity of copper, *ρ*, is 2.3 × 10^−6^ Ω-m. Thus, resistance of the primary winding is calculated by

(18) $$R_{MPP}\;=\;\rho \frac{l}{A}\;=\;2.3\;\times \;10^{-6}\;\frac{894.4}{0.518}\;=\;3.971\;m\Omega$$

Suppose that the turns ratio of coupled inductor is unity. The total copper loss is calculated as

(19) $$P_{copper-}{}_{MPP}\;=\;9.33^{2}\;\times \;3.971\;\times \;2\;=\;691.17\;\mathrm{mW}$$

### 5.2. High Flux Core

With respect to the core loss of the magnetic material of High Flux, it can be estimated by

(20) $$P_{core\;loss-High\;Flux}\;=\;246\;B^{2.23}f_{s}{}^{1.47}$$

**Figure 5:**
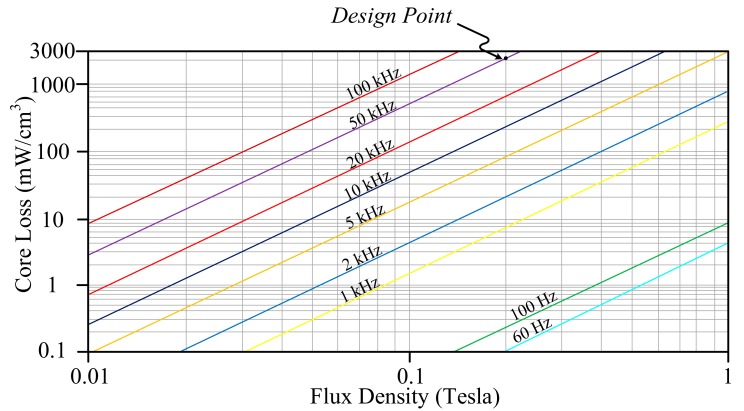
The relationship between core loss and flux density of High Flux, while permeability equals 125 μH/m.

Figure 5 illustrates the relationship of core loss versus flux density under different switching frequencies, while permeability is 125 μH/m. If 0.2-Tesla flux density is the operation point and the core of High Flux 58324-A2 is chosen, then the associated core loss density will be 2136.57 mW/cm^3^ under 50-kHz switching frequency. Since the volume of the core 58324-A2 is 6.088 cm^3^, core loss of the High Flux magnetic coupled inductor is 13.007 W.

For High Flux 58324-A2, the allowed maximum variation at flux density is up to 0.75 Tesla. With the same conditions in MPP, the turns of primary side can be determined as follows:

(21) $$N_{\mathrm{High}\;\mathrm{Flux}}\;=\;\frac{L_{m1}\Delta I_{max}}{\Delta B_{max}A_{e}}\;\times \;10^{-2}\;=\;\frac{70.4\times 9.33}{0.75\times 0.678}\;\times \;10^{-2}\;=\;12.91$$

Since turns ratio should be an integer, *N_High Flux_* is chosen as 13 turns. An average length of one turn is 3.44 cm for High Flux 58324-A2. Therefore, the total length of the winding at the primary is

(22) $$l_{\mathrm{High}\;\mathrm{Flux}}\;=\;13\;\times \;3.44\;=\;447.2\;\mathrm{mm}$$

Resistance of the primary winding is calculated as

(23) $$R_{\mathrm{High}\;\mathrm{Flux}}\;=\;\rho \frac{l}{A}\;=\;2.3\;\times \;10^{-6}\;\frac{447.2}{0.518}\;=\;1.985\;m\Omega$$

Then, the total copper loss of the coupled inductor with High Flux core is

(24) $$P_{copper-\mathrm{High}\;\mathrm{Flux}}\;=\;9.33^{2}\;\times \;1.985\;\times \;2\;=\;345.58\;\mathrm{mW}$$

### 5.3. Kool Mμ Core

Another magnetic core widely adopted in switching power circuit is the type of Kool Mμ. Its core loss can be determined by the following equation.

(25) $$P_{core\;loss-\mathrm{Kool}\;M\mu }\;=\;91.58\;B^{2.2}f_{s}{}^{1.63}$$

**Figure 6:**
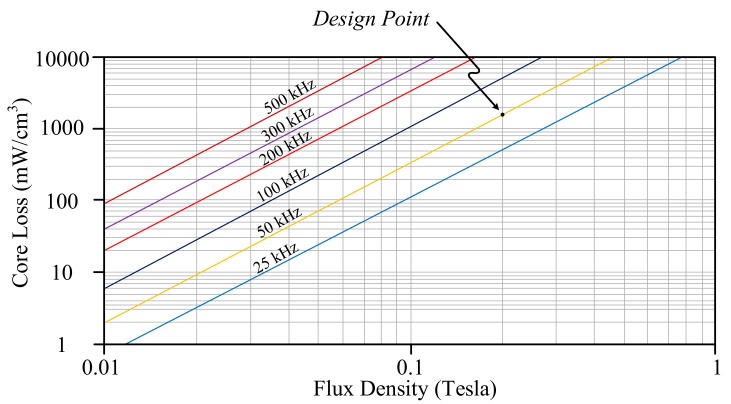
The relationship between core loss and flux density of Kool Mμ, while permeability equals 125 μH/m.

Based on (25), a corresponding plot is presented in Figure 6, while permeability equals 125 μH/m. A Kool Mμ core with part no. 77324-A2 is chosen, whose volume is 6.088 cm^3^. Its associated power loss density will be 1560.94 mW/cm^3^ under the conditions, *B* = 0.2 Tesla and *f_s_* = 50 kHz, which results in a core loss of 9.503 W.

The maximum variation of flux density of Kool Mμ is 0.525 Tesla. Similarly, with the same given values as in the discussions of MPP and High Flux cores, the turns at the primary winding is determined as

(26) $$N_{\mathrm{Kool}\;M\mu }\;=\;\frac{L_{m1}\Delta I_{max}}{\Delta B_{max}A_{e}}\;\times \;10^{-2}\;=\;\frac{70.4\times 9.33}{0.525\times 0.678}\;\times \;10^{-2}\;=\;18.45$$

Thus, *N*_Kool Mμ_ is chosen as 18 turns. Because an average length of one turn is around 3.44 cm, the total length of the primary winding is 619.2 mm. The resistance of the primary winding is calculated as

(27) $$R_{\mathrm{Kool}\;M\mu }\;=\;\rho \frac{l}{A}\;=\;2.3\;\times \;10^{-6}\;\frac{619.2}{0.518}\;=\;2.749\;m\Omega$$

In addition, the copper loss of a coupled inductor with unity turns ratio can be estimated as

(28) $$P_{copper-\mathrm{Kool}\;M\mu }\;=\;9.33^{2}\;\times \;2.749\;\times \;2\;=\;478.59\;\mathrm{mW}$$

## 6. Experimental Results

To validate the proposed SIUSC, a 200-W prototype is built with the specifications and components that are summarized in Table 1, while the measuring equipment are shown in Table 2. In the experiment, the input and output voltages are 24 and 400 V, respectively. The implementation of the coupled inductors *T*_1_ and *T*_2_ are based on MPP 55324-A2 core with a turns ratio of 1:1, which have magnetizing inductances 70.4 and 70.6 μH, respectively, which are measured by MICRTEST 6377 (MICROTEST Corp., Taipei, Taiwan). Current-mode PWM IC, UC3843 (TEXAS INSTRUMENTS, Dallas, TX, USA), is chosen as converter controller. The power MOSFET IRFP4332PbF (INTERNATIONAL RECTIFIER, El Segundo, CA, United States) with 200-mΩ on-state resistance of *R_ds(on)_* is served as main switch. Power Schottky Rectifier SBL6045PT, of which the forward voltage is 0.44 V, is in charge of *D*_1_ and DSSK60-02AR with 0.7-V forward voltage is for *D*_2_, *D*_3_ and *D_lk_*. In addition, ultrafast power diode BYV34 is used for *D*_4_, *D*_5_, and *D_o_*, which has a forward voltage of 1.05 V. The input of the prototype is powered by the voltage power supply, IDRC CDSP-060-100C (IDRC, Taipei, Taiwan). Meanwhile, the converter output is connected to an electronic load, GW Instek PEL-2041 (GWINSTEK, Taipei, Taiwan). All of the waveforms are measured by oscilloscope KEYSIGHT DSO-X 3024A (KEYSIGHT, Santa Rosa, CA, USA).

**Figure 7:**
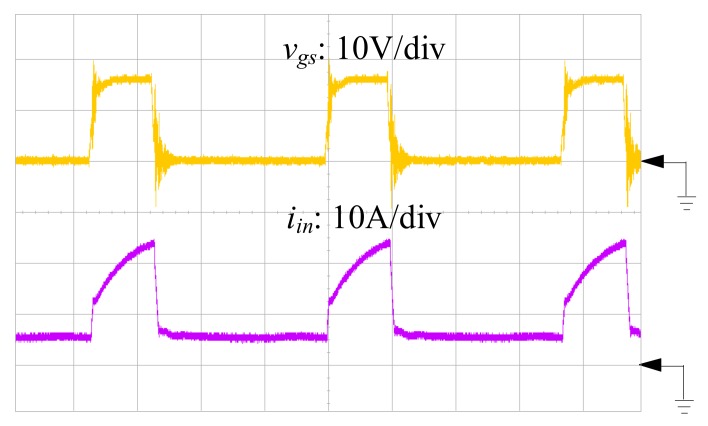
The measured waveforms of control signal *v_gs_* and input current *i_in_*.

**Figure 8:**
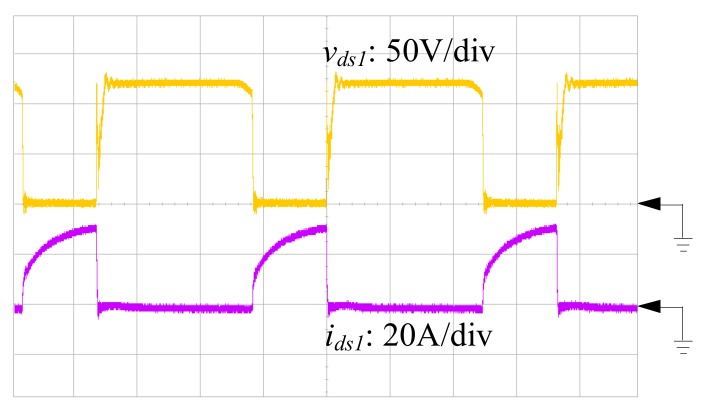
The measured waveforms from active switch *S*_1_.

**Figure 9:**
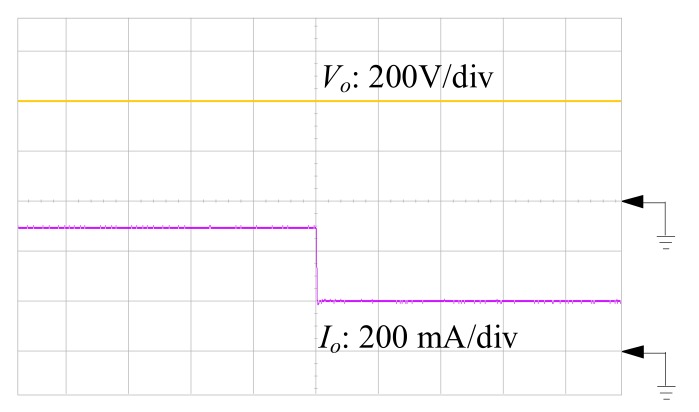
Measured output voltage and current waveforms to illustrate transient response under step change.

**Figure 10:**
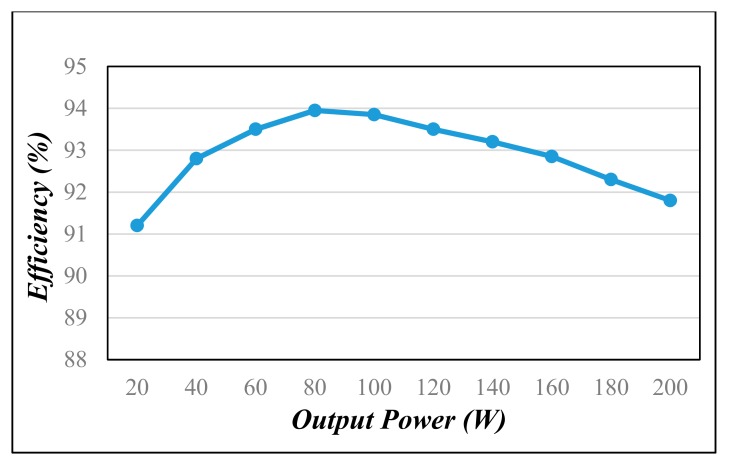
The measured efficiency of the proposed converter.

**Figure 11:**
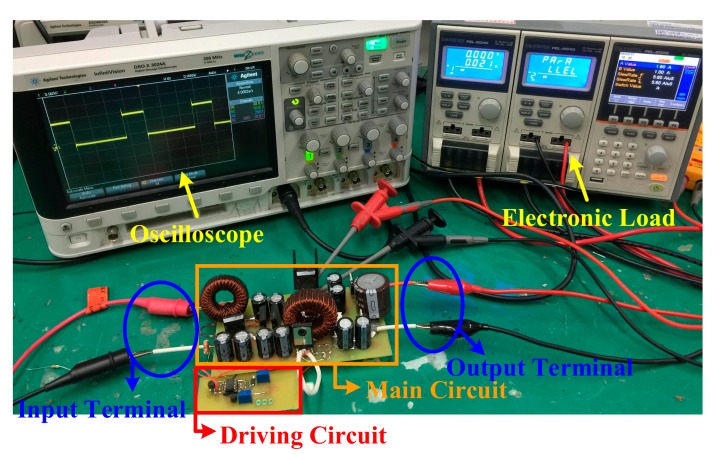
The photo of the experimental setup.

Figure 7, Figure 8 and Figure 9 are the practical measurements. Figure 7 illustrates the input current of the proposed converter, which verifies that the SIUSC indeed operates in CCM. Figure 8 shows the measurements from active switch *S*_1_, in which it can be observed that the *v_ds_*_1_ can be clamped to a lower voltage stress. This voltage-clamped effect also demonstrates the feature of leakage energy recycling. Figure 9 shows the transient response of the converter under step load change, which confirms that the SIUSC can intrinsically achieve excellent performance. Figure 10 depicts the measured efficiency of the prototype, where the peak efficiency is around 94% at 80 W. Figure 11 shows the photo of test bench, where the equipment for practical measurement have been listed in Table 2. In Figure 11, the length, width, and height of the main circuit are around 13.8, 8.04, and 3.4 cm, respectively.

## 7. Conclusions

This paper proposes a novel single-switch isolated ultra-high step-up DC/DC converter, which is applicable to fuel cells, PV module, and battery system. The main contribution of this paper is that an ultra-high voltage conversion ratio can be easily achieved even under a low turns ratio and duty ratio. This outstanding performance make the SIUSC be much more suitable for any low voltage source to boost its input voltage. In addition, the loss of magnetic components is assessed and discussed for three different types of toroidal cores. A 24/400 V 200 W prototype has been built and examined to verify the feasibility of the SIUSC. The measurements indicate the converter having a maximum efficiency of 94%.

## Figures and Tables

**Table 1:** Specifications and components used in experiments.

Symbols	Values & Types
*V_in_* (input voltage)	24 V
*V_o_* (Output Voltage)	400 V
*P_o_* (output power)	200 W
*f_s_* (switch frequency)	50 kHz
*n*_1_, *n*_2_ (transformer turns ratio)	1
*L_m_*_11_ (magnetizing inductance)	70.4 μH
*L_m_*_21_ (magnetizing inductance)	70.6 μH
*L_lk_*_11_ (Leakage inductance)	1.45 μH
*L_lk_*_21_ (Leakage inductance)	1.45 μH
*C*_1_, *C*_2_, *C*_3_ (Capacitance)	47 μF
*C_lk_* (Capacitance)	5 μF
*C*_4_, *C*_5_ (Capacitance)	100 μF
*C_o_* (Capacitance)	220 μF
*S*_1_ (Switch)	IRFP4332PbF
*D*_1_	SBL6045PT
*D*_2_, *D*_3_, *D_lk_* (Diodes)	DSSK60-02AR
*D*_4_, *D*_5_, *D_o_* (Diodes)	BYV34

**Table 2:** Measurement Equipment.

Current Probe	Tektronix TCP312, 30ADL
Power Supply	iDRC CDSP-060-100C
Oscilloscope	Agilent DSO-X 3024A 200MHz 4GSa/s
DC Load	GW Instek PEL-2041 0 A/10 A 2.5 V/500 V 350 W
LCR Meter	MICROTEST 6377
